# MB-SwinRefiner: Mammographic Mass Segmentation with Probability-Guided Residual Refinement

**DOI:** 10.3390/jimaging12080361

**Published:** 2026-08-07

**Authors:** Zainab Shanta Swayedi, Pedram Salehpour, Hadi Aghdasi

**Affiliations:** Faculty of Electrical and Computer Engineering, University of Tabriz, Tabriz 5166616471, Iranaghdasi@tabrizu.ac.ir (H.A.)

**Keywords:** mammographic mass segmentation, deep learning, swin transformer, multi-scale decoder fusion, probability-guided residual refinement

## Abstract

Accurate mammographic mass segmentation is crucial for computer-aided breast cancer diagnosis, but remains challenging because masses may be small, low-contrast, irregularly shaped, and partially obscured by dense fibroglandular tissue. Although recent methods have improved contextual representation and multi-scale feature extraction, reliable segmentation still requires better integration of lesion-scale representation, contour-derived auxiliary supervision, and local probability-map refinement. This paper proposes MB-SwinRefiner, a novel two-stage framework consisting of a Multi-scale Boundary-aware SwinUNet (MB-SwinUNet) stage and a probability-guided residual refinement stage. The first stage, MB-SwinUNet, generates an initial mass probability map using hierarchical Swin encoding, multi-scale decoder fusion, and training-only auxiliary boundary supervision. The second stage refines this prediction through probability-guided residual correction by using the Stage 1 probability map as a prior and learning logit-space corrections. Experiments were conducted on a case-level five-fold cross-validation of the INbreast dataset under both a resized benchmark setting and a native sliding-window full-mammogram setting. Performance was evaluated using Dice, intersection over union, sensitivity, and specificity. In the resized benchmark setting, MB-SwinRefiner achieved Dice of 86.19%, IoU of 84.33%, sensitivity of 89.34%, and specificity of 99.98%. In the native sliding-window setting, MB-SwinRefiner improved mean Dice from 76.64% to 78.06% and mean IoU from 74.38% to 76.11%. These results suggest that combining Swin-based contextual encoding, multi-scale decoder fusion, contour-derived training supervision, and probability-guided residual refinement can improve region-based mammographic mass segmentation performance.

## 1. Introduction

Mammography remains the principal imaging modality for breast cancer screening [1]. However, accurate mass segmentation in full-field mammograms remains challenging because masses often occupy only a small fraction of the image, may have weak contrast against surrounding tissue, and can be partially obscured by dense fibroglandular parenchyma [2,3,4]. This difficulty is clinically important because dense breast tissue can reduce mammographic sensitivity by masking lesions, contributing to missed cancers and interval cancers [2,3,4]. In addition to lesion-to-background ambiguity, mammographic mass segmentation must address severe class imbalance, substantial variation in mass size and morphology, and clinically relevant boundary patterns such as indistinct, irregular, or spiculated margins [5]. Recent mammography studies further indicate that mass area can range from well below 1% of the image to much larger fractions across samples, making robust segmentation a joint problem of contextual disambiguation, scale-aware reconstruction, and contour localization rather than a simple foreground–background classification task [5].

Deep learning methods for mammographic mass segmentation have been developed under different input and evaluation protocols, including mass-centered patches, detected regions of interest, resized full mammograms, and full-image inference. Resized image-level evaluation is commonly used because it provides a practical benchmark setting and enables comparison with previously reported results. However, resizing the whole mammogram can also alter the apparent lesion scale, boundary appearance, and foreground-to-background relationship. Therefore, both settings are informative: the resized setting is useful for benchmarking against prior work, while native sliding-window inference is useful for analyzing how a model behaves when predictions are reconstructed at the original mammogram scale.

Because mammographic mass segmentation is strongly affected by lesion–background ambiguity, recent methods have increasingly moved beyond purely convolutional designs toward architectures that more explicitly model broader contextual dependencies. CNN-based methods have strengthened representation learning through attention, multi-level pyramids, contextual affinity, adaptive receptive fields, and skip redesign [6,7,8,9,10,11,12,13,14,15], whereas transformer and hybrid mammographic models have introduced token-based or cross-attention contextual encoding, including YOLO-LOGO, TrEnD, Swin-SFTNet, HTU-Net, and Mammo-SAM [16,17,18,19,20]. In the broader medical segmentation literature, transformer-family models such as TransUNet, TransFuse, Swin-Unet, nnFormer, and UNETR have also shown that attention-based contextual encoding can provide strong representations for dense prediction [21,22,23,24,25]. These studies suggest that hierarchical contextual encoding is valuable for mammographic segmentation, but they also show that accurate pixel-level mass delineation still depends on how the encoded features are reconstructed, regularized, and refined.

Decoder design remains important because contextual features must ultimately be reconstructed into spatially precise mass masks. Prior medical and mammographic segmentation studies have shown the value of multi-scale feature aggregation and improved skip-connected reconstruction [7,12,13,14,15,19,26,27,28]. However, the final prediction may still rely disproportionately on the last decoder representation. This motivates the proposed fusion of reconstructed decoder features from multiple semantic levels before final mask prediction.

Moreover, region-overlap losses may not fully preserve structural information around weak or irregular mass contours. Previous work has therefore investigated contour-sensitive losses, auxiliary supervision, and dedicated edge or shape pathways [5,29,30,31,32,33,34,35,36,37]. In this study, contour-derived targets are used only as auxiliary training supervision to regularize the shared decoder representation, while the boundary branch is removed during inference.

Motivated by these observations, we propose MB-SwinRefiner, a mammographic mass segmentation framework built around a Multi-scale Boundary-aware SwinUNet (MB-SwinUNet) and a probability-guided residual refinement stage. MB-SwinUNet uses a hierarchical Swin encoder, decoder-side multi-scale fusion, and training-only auxiliary boundary supervision to generate mass probability maps. To support comparison with prior work, MB-SwinUNet is first evaluated under a commonly used resized 512 × 512 image-level setting. The framework is then analyzed under a native sliding-window full-mammogram setting, where patch-level predictions are aggregated into a full-image probability map. MB-SwinRefiner uses the fixed Stage 1 probability map to guide decoder reconstruction and predicts an additive correction to the Stage 1 logits rather than an independent replacement mask. Although the training-only boundary branch does not add inference-time computation, the complete two-stage framework requires an additional refinement pass compared with Stage 1 alone. More broadly, deep medical-image segmentation networks may involve substantial parameter counts and computational requirements, motivating continued investigation of efficient training and model-compression strategies such as knowledge distillation [38].

The main contributions of this work are fourfold:

1. Full-mammogram mass segmentation is challenged by extreme foreground sparsity, scale variation, and the loss of lesion detail that may occur when whole mammograms are resized to a fixed low resolution. To address this, we propose MB-SwinUNet, a Swin-based mammographic mass segmentation model that uses multi-scale decoder fusion to combine reconstructed decoder features before final prediction.
2. Region-overlap losses alone may not fully preserve structural information around weak or irregular mammographic margins. We therefore introduce a training-only auxiliary boundary branch that regularizes the shared decoder features using contour-derived supervision without adding inference-time complexity.
3. To analyze full-mammogram segmentation at the original image scale, we extend MB-SwinUNet to native sliding-window inference, where patch-level predictions are aggregated into full-image probability maps. This setting preserves the native mammogram scale and allows the effects of multi-scale decoder fusion and boundary supervision to be evaluated under full-image reconstruction.
4. We formulate Stage 2 refinement as probability-guided logit residual correction. The fixed Stage 1 probability map modulates selected decoder features, while a zero-initialized correction head predicts additive changes to the Stage 1 logits. This design constrains the refiner to learn corrections to the initial prediction rather than independently generating a replacement mask. The same formulation is applied to both resized images and spatially aligned native sliding-window patches.

The remainder of this paper is organized as follows. Section 2 reviews related work, Section 3 describes the materials and methods, Section 4 presents the experimental results, and Section 5 and Section 6 provide the discussion and conclusion, respectively.

## 2. Related Work

### 2.1. Contextual Reasoning in Mammographic Mass Segmentation

A major direction in mammographic mass segmentation has shifted from patch-centered or region-of-interest-dependent pipelines toward whole-mammogram segmentation, because localization errors introduced upstream cannot always be recovered by the downstream segmentation model. In full-field mammograms, sparse and weakly contrasted lesions must be distinguished from fibroglandular tissue, overlapping structures, and density-related patterns that may resemble masses. CNN-based methods have addressed this challenge through attention-guided dense upsampling, adaptive channel–spatial context modeling, contextual affinity learning, and adaptive receptive-field design [6,7,8,9,10]. Collectively, these studies established that mass delineation depends not only on local edge information but also on broader lesion–background context.

CNN-based mammographic segmentation methods have generally followed two complementary directions: strengthening contextual representation through attention, affinity modeling, and adaptive receptive fields, and improving multi-level feature transfer through pyramid, nested, or cascaded encoder–decoder structures [6,7,8,9,10,11,12,13,14,15]. Although these approaches have achieved strong results, their broader contextual modeling remains largely mediated by convolutional operations. This limitation is particularly relevant in dense mammograms, where lesions may resemble the surrounding parenchyma over extended regions, motivating architectures that model hierarchical contextual dependencies more explicitly.

This development should not be interpreted as replacing CNNs with transformers. CNNs remain effective for extracting local texture, low-level gradients, and fine boundary cues, particularly for irregular, indistinct, or spiculated masses. Transformer and hybrid architectures instead complement these local representations by providing broader contextual modeling through transformer modules, hybrid CNN–Transformer encoders, and cross-attention mechanisms [16,17,18,19,20]. The broader trend is therefore toward architectures that combine local contour evidence with global lesion–background context.

### 2.2. Transformer and Foundation-Model Segmentation: Stronger Context, Unresolved Spatial-Detail Recovery

Transformer-based segmentation models were introduced into medical imaging to model long-range dependencies beyond the locality of standard convolutions. TransUNet combined transformer-based global context with U-shaped decoding and CNN skip features for localization [21], while TransFuse explicitly fused transformer representations with convolutional local features [22]. Swin-Unet organized hierarchical shifted-window attention within a U-shaped architecture [23], and models such as nnFormer and UNETR further demonstrated the value of attention-based contextual modeling when combined with appropriate skip connections and hierarchical decoding [24,25]. Recent frequency-aware directional-shift methods have similarly explored efficient long-range interaction and edge-sensitive representation [39]. Collectively, these studies show that strong contextual encoding must be accompanied by effective spatial reconstruction.

Transformer-oriented mammographic segmentation has followed a similar direction. YOLO-LOGO introduced a transformer-based framework for breast-mass detection and segmentation [16], while TrEnD used adaptive patch embedding to address variation in lesion size and appearance [17]. Swin-SFTNet emphasized spatial feature expansion and aggregation for whole-breast micro-mass segmentation [18], and HTU-Net combined CNN and transformer components through channel–spatial representation and cross-attention fusion [19]. These methods demonstrate the value of transformer-based context in mammography, while their reliance on feature expansion, skip refinement, and hybrid fusion indicates that encoder-side context alone is insufficient for recovering pixel-level mass structure.

Hierarchical transformer encoders increase semantic abstraction through token mixing, patch merging, and window-based attention. Although these mechanisms help distinguish lesions from surrounding parenchyma, repeated downsampling and semantic smoothing may weaken high-frequency structures associated with spiculated, lobulated, or low-contrast margins. Consequently, the effectiveness of Swin-based mammographic segmentation depends not only on contextual encoding but also on the reconstruction, regularization, and refinement of hierarchical features at pixel resolution.

Foundation models further illustrate the need for task-specific adaptation. SAM introduced a large-scale promptable segmentation paradigm [40], while MedSAM and SegVol extended this direction to medical and volumetric imaging [41,42]. However, zero-shot transfer remains inconsistent for targets with weak boundaries, subtle contrast, and modality-specific appearance [43]. Mammo-SAM therefore adapted SAM specifically for automatic mass segmentation in whole mammograms [20]. These findings indicate that large-scale contextual representations still require modality-aware decoding and task-specific optimization for mammographic segmentation.

### 2.3. Decoder Fusion Beyond Conventional Skip Aggregation

Decoder design remains a major determinant of segmentation quality because contextual features must ultimately be converted into spatially precise masks. UNet++ redesigned skip pathways through nested dense connections to reduce the semantic gap between encoder and decoder features [26], while UNet 3+ introduced full-scale feature aggregation and deep supervision to combine low-level detail with high-level semantics across multiple resolutions [27]. More recent decoder-centric architectures, such as EMCAD, have further demonstrated the value of efficient multi-scale attention decoding, even when strong encoders are available [28]. These developments establish multi-scale decoding as an important design principle; the remaining methodological question concerns where fusion is performed and which representations are combined.

Most nested and full-scale architectures aggregate encoder- and decoder-derived features across multiple resolutions during reconstruction. The proposed strategy instead fuses selected reconstructed decoder-stage representations immediately before final mask prediction. These representations capture complementary information: deeper decoder features preserve coarse lesion extent and contextual evidence, whereas shallower features retain finer spatial and peripheral cues. Combining them before prediction may therefore reduce excessive dependence on the final decoder layer and improve the reconciliation of lesion extent and boundary structure.

This distinction is particularly relevant to UNet 3+, which provides decoder layers with multi-resolution features through full-scale skip pathways. In contrast, the proposed fusion operates on decoder representations after they have undergone Swin-based contextual encoding and progressive reconstruction. The objective is not simply to aggregate raw or skip-transferred multi-scale features, but to combine reconstructed lesion representations corresponding to different semantic and spatial levels. Thus, the proposed approach complements established full-scale aggregation by focusing specifically on the reconciliation of coarse lesion extent and fine peripheral evidence immediately before prediction.

Mammography-specific architectures further support the importance of decoder reconstruction. AUNet introduced attention-guided dense upsampling [7], FS-UNet strengthened mass-relevant encoder–decoder representations [12], and Connected-UNets, Connected-SegNets, and EU-Net improved reconstruction through connected, cascaded, or enhanced U-shaped pathways [13,14,15]. More recently, HTU-Net combined hybrid transformer encoding with multiscale cross-attention fusion [19]. Collectively, these methods confirm the importance of decoder and skip-connection design while indicating that scale-consistent reconstruction remains challenging under dense-tissue ambiguity and heterogeneous mass morphology. Decoder-stage fusion over reconstructed representations is therefore proposed as a targeted extension of existing multi-scale reconstruction strategies.

### 2.4. Boundary-Aware Optimization and Training-Time Contour Regularization

Boundary-aware segmentation has been widely studied to complement region-overlap objectives with contour-sensitive constraints. Boundary Loss incorporates distance-to-boundary information to address highly imbalanced segmentation [29], while Hausdorff-distance-based losses penalize large contour deviations that may not be adequately reflected by overlap metrics [30]. Gated-SCNN introduced a dedicated shape stream for contour refinement [31], and BEA-Net separated body and edge representations through multi-scale body–edge-aware decoding [32]. Active boundary loss and boundary-uncertainty formulations further demonstrate the shift from purely region-based objectives toward contour-sensitive optimization [33,34]. Collectively, these approaches show that Dice- and cross-entropy-based objectives do not fully constrain localized boundary errors.

This limitation is particularly relevant in mammography, where clinically meaningful errors often occur along weak, irregular, or spiculated margins. A model may achieve acceptable Dice or IoU while missing thin spiculations, smoothing indistinct contours, or extending into adjacent dense tissue. Mammography-specific studies have therefore explored deep supervision, adversarial segmentation, density- and size-aware losses, and auxiliary-task learning to improve robustness under class imbalance and ambiguous lesion appearance [5,35,36,37]. However, these approaches vary in how directly they supervise boundary structure and whether they require additional inference-time components. It is therefore desirable to incorporate contour-derived supervision without relying on iterative post-processing or a permanent parallel edge pathway.

In the proposed framework, the boundary branch is formulated as training-time structural regularization rather than as an independent inference pathway. It is attached to the shared decoder representation and optimized using a contour-derived auxiliary target, while the final segmentation mask is produced by the main segmentation head. Because the boundary prediction is neither fed into the segmentation head nor used to construct the final mask, the branch can be removed during inference without changing the deployed segmentation pathway. Its purpose is to provide additional gradients that encourage contour-sensitive decoder features.

This design differs from persistent shape- or edge-stream approaches such as Gated-SCNN [31] and BEA-Net [32]. It does not provide an explicit boundary pathway at inference; instead, it introduces contour-derived regularization without additional inference-time computation. The contribution of this supervision and its interaction with decoder-stage multi-scale fusion are evaluated through the ablation experiments described in Section 4.

Table 1 summarizes representative mammographic and medical segmentation approaches, their principal design strategies, and the remaining limitations addressed by the proposed framework.

## 3. Materials and Methods

The proposed framework is built around a Swin-based [44] encoder–decoder segmentation architecture. In the first stage, MB-SwinUNet generates an initial mammographic mass probability map. The Swin encoder extracts hierarchical contextual features through shifted-window self-attention, while the decoder restores spatial resolution through skip-connected reconstruction. Because mammographic masses may be small, low-contrast, and weakly bounded, the segmentation pathway should integrate coarse lesion context with higher-resolution spatial features.

To address this requirement, MB-SwinUNet integrates multi-scale decoder fusion and training-only auxiliary boundary supervision. Multi-scale decoder fusion combines reconstructed decoder features from different semantic levels before final prediction, allowing the segmentation head to use coarse, intermediate, and fine spatial information jointly. The auxiliary boundary branch encourages contour-sensitive feature learning during training and is removed during inference, avoiding additional deployment complexity.

The second stage, MB-SwinRefiner, uses the Stage 1 probability map as prior information for probability-guided residual refinement. Instead of re-segmenting the mammogram independently, it learns logit-space corrections from the mammogram input and the corresponding Stage 1 probability map. The framework is evaluated in both a resized setting and a sliding-window full-mammogram setting, enabling comparison under a common resized protocol and analysis of full-image reconstruction at the original mammogram scale.

### 3.1. MB-SwinUNet for Resized and Native Sliding-Window Segmentation

**Figure 1:**
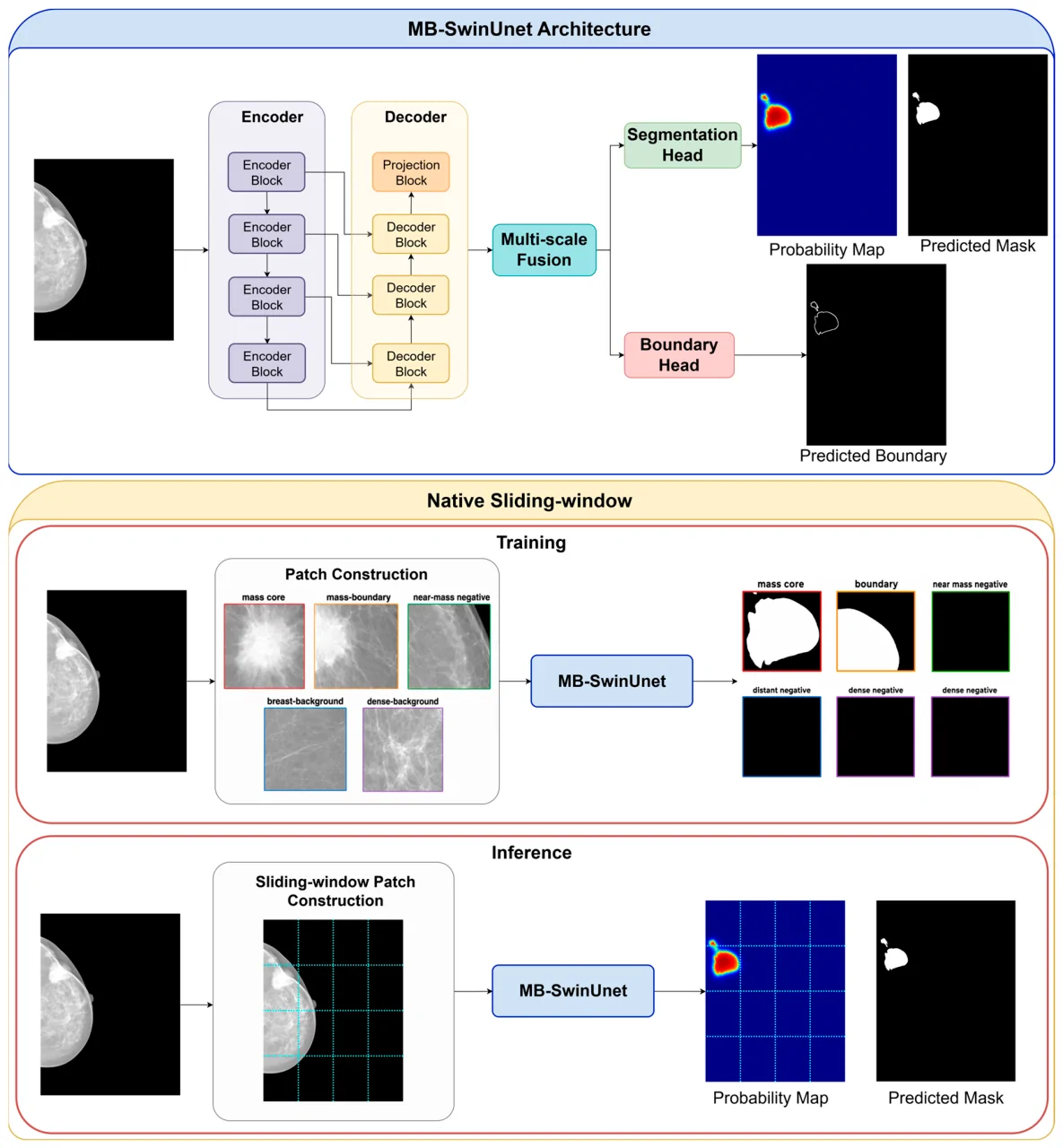
Stage 1 MB-SwinUNet architecture and native sliding-window inference. Multi-scale decoder features are fused to generate a mass probability map, while the auxiliary boundary branch is used only during training. Overlapping patch predictions are averaged to reconstruct the full-mammogram probability map and binary mask.

The first stage of the proposed framework is MB-SwinUNet (shown in Figure 1), a Swin-based encoder–decoder segmentation network designed to generate an initial mammographic mass probability map. In this work, MB denotes Multi-scale Boundary-aware, referring to the combination of multi-scale decoder fusion and training-only auxiliary boundary supervision. The same Stage 1 architecture is used in two experimental settings. In the resized benchmark setting, the full mammogram and its corresponding mask are resized to 512 × 512, and MB-SwinUNet predicts the mass probability map directly at the resized image level. In the native sliding-window setting, the network operates on local 512 × 512 mammogram patches and the patch-level predictions are aggregated to reconstruct a full-image probability map at the original mammogram scale.

Given an input $x\in R^{1\times 512\times 512}$, where x denotes either a resized full mammogram or a native mammogram patch, MB-SwinUNet predicts a mass logit map $z^{\left(1\right)}\in R^{C\times H\times W}$, and the corresponding probability map is obtained as $p^{\left(1\right)}=\sigma \left(z_{p}^{\left(1\right)}\right)$, where $\sigma \left(\cdot \right)$ denotes the sigmoid function. The input has one channel because mammograms are grayscale images. Therefore, the Swin patch-embedding layer is configured with in_chans = 1. When ImageNet-pretrained weights [45] are used, the RGB patch-embedding weights are converted to a single-channel kernel by averaging across the input-channel dimension:

(1) $$W_{gray}\left(o,1,i,j\right)=\frac{1}{3}\sum_{c=1}^{3}W_{RGB\;}\left(o,c,i,j\right).$$

This produces a grayscale patch-embedding kernel while preserving the pretrained spatial projection filters. Mammograms were scaled and normalized using the parameters described in Section 3.3.

The encoder is implemented using a Swin-Tiny backbone in feature-extraction mode. Although Swin-Tiny is originally pretrained on natural images, the architecture can be applied to 512 × 512 mammographic inputs because it relies on local shifted-window self-attention and relative position bias rather than a fixed global absolute position embedding tied to the original pretraining resolution. The relative position bias is associated with the local attention window and is therefore retained during fine-tuning. For an input, the encoder produces four hierarchical feature maps:

$$E_{1}\in R^{96\times 128\times 128},E_{2}\in R^{192\times 64\times 64},E_{3}\in R^{384\times 32\times 32},E_{4}\in R^{768\times 16\times 16}.$$

These feature maps correspond to spatial scales of 1/4, 1/8, 1/16, and 1/32 of the input resolution. The deepest feature map provides a coarse contextual representation of the lesion and surrounding tissue, while the shallower feature maps retain finer spatial information that is useful for boundary localization.

The decoder is a skip-connected convolutional reconstruction path that progressively restores spatial resolution from the deepest encoder feature. Starting from $E_{4}$, each decoder stage performs bilinear upsampling, 1×1 channel projection, channel-wise concatenation with the corresponding encoder skip feature, and residual convolutional refinement. The first decoder stage upsamples $E_{4}$ to the spatial size of $E_{3}$, projects the feature channels, concatenates the result with $E_{3}$, and refines the combined tensor to produce

$$D_{3}\in R^{384\times 32\times 32}.$$

The same reconstruction procedure is repeated at the next two scales, producing

$$D_{2}\in R^{192\times 64\times 64},\;\;\;\;\;D_{1}\in R^{96\times 128\times 128}.$$

After the final skip-connected decoder stage, $D_{1}$ is bilinearly upsampled to the full input resolution, projected to 64 channels, and refined to produce the full-resolution decoder feature

$$D_{f}\in R^{64\times 512\times 512}.$$

Each residual refinement block consists of a 1 × 1 projection followed by two 3 × 3 convolutional layers with group normalization and GELU activation. Let *X* denote the input to a residual refinement block. The block can be written as

(2) $$X^{\prime }=Conv_{1\times 1}\left(X\right),\;$$

(3) $$R(X)=GELU\left(X^{\prime }+Conv_{3\times 3}^{\left(2\right)}\left(GELU\left(Conv_{3\times 3}^{\left(1\right)}\left(X^{\prime }\right)\right)\right)\right)$$

Group normalization [46] is applied after each convolutional projection before activation. It is used instead of batch normalization because training is performed with a small effective batch size, where batch statistics may be unstable.

The main reconstruction component of MB-SwinUNet is multi-scale decoder fusion (MSDF). Unlike a decoder that predicts the segmentation mask only from the final decoder feature, MSDF combines reconstructed decoder features from multiple semantic levels before final mask prediction. In the implemented configuration, the selected fusion levels are $\left\{D_{3},D_{2},D_{f}\right\}$.

The feature $D_{1}$ is not used as a separate fusion input because it is immediately transformed into $D_{f}$ through full-resolution upsampling and refinement. Including both $D_{1}$ and $D_{f}$ would introduce a redundant high-resolution pathway. Therefore, MSDF combines one deeper decoder representation, one intermediate decoder representation, and the final full-resolution decoder representation. This design allows the final predictor to use coarse lesion context, mid-level structural information, and fine spatial localization jointly.

Each selected decoder feature is first projected to 64 channels using a 1×1 convolution. Lower-resolution features are then bilinearly upsampled to the full resolution:

(4) $${\tilde{D}}_{k}=U_{k}\left(Conv_{1\times 1}\left(D_{k}\right)\right),\;\;\;\;\;\;k\in \left\{3,2,f\right\},\;$$

where $U_{k}\left(\cdot \right)$ denotes bilinear upsampling to the full input resolution when required. The aligned features are concatenated and passed through a residual fusion block:

(5) $$F^{\left(1\right)}=R_{MSDF}\left(Concat\left({\tilde{D}}_{3},{\tilde{D}}_{2},{\tilde{D}}_{f}\right)\right),$$

where $F^{\left(1\right)}\in R^{64\times 512\times 512}$.

Although MSDF operates on decoder-level features, these representations remain encoder-informed because $D_{3}$, $D_{2}$, and $D_{1}$ are generated through skip-connected reconstruction from the hierarchical Swin encoder features. The fused representation therefore combines contextual encoding with reconstructed spatial detail.

The fused representation $F^{\left(1\right)}$ is shared by the segmentation and auxiliary boundary heads. The segmentation head consists of a 3×3 convolutional refinement layer followed by a 1×1 projection, producing the Stage 1 mass logit map:

(6) $$z_{p}^{\left(1\right)}=H_{seg}\left(F^{\left(1\right)}\right),$$

The probability output is then obtained as

(7) $$p_{p}^{\left(1\right)}=\sigma \left(z_{p}^{\left(1\right)}\right)$$

In parallel, the boundary head uses two 3×3 convolutional layers followed by a 1×1 projection to predict a boundary logit map:

(8) $$b_{p}^{\left(1\right)}=H_{bnd}\left(F^{\left(1\right)}\right),$$

The boundary target is generated from the binary mass mask using a morphological-gradient operation. Given a binary mass mask *Y*, the boundary target is defined as

(9) $$Y_{b}^{bnd}=Dilate\left(Y_{p};K_{10}\right)-Erode\left(Y_{p};K_{10}\right),$$

where $K_{10}$ is a 10×10 all-ones kernel. This produces a boundary band around the annotated mass contour and provides an additional structural training signal to the shared decoder representation. The auxiliary boundary branch is removed during inference and therefore does not increase the cost of either individual stage. However, the complete two-stage framework requires an additional refinement pass compared with Stage 1 alone.

The Stage 1 loss is defined directly from the segmentation and boundary outputs.

(10) $$L_{stage1}={\omega_{1}L}_{focal}^{seg}+{\omega_{2}L}_{tversky}^{seg}+{\omega_{3}L}_{focal}^{bnd}$$

The segmentation loss combines focal loss and Tversky loss to address foreground sparsity and class imbalance, while the boundary focal loss provides additional contour-aware supervision [47,48].

During inference, the output protocol depends on the experimental setting. In the resized benchmark setting, the full mammogram is resized to 512×512, passed through MB-SwinUNet once, and the predicted probability map is thresholded to obtain the final binary segmentation at the resized image level. This setting is used to provide comparison with prior studies that evaluate INbreast segmentation using resized mammographic inputs.

In the native sliding-window setting, MB-SwinUNet is applied to the full preprocessed mammogram using overlapping sliding windows. The inference stride is set to 256 pixels, corresponding to 50% overlap between adjacent windows. For a window located at coordinate $\left(x,y\right)$, the model predicts a probability patch $p_{x,y}^{\left(1\right)}$. The set of window coordinates includes the last valid positions along both axes, ensuring complete image coverage. If an image dimension is smaller than the patch size, reflection padding is applied to complete the input patch, and only the valid non-padded region is retained after prediction.

The full-image Stage 1 probability map is obtained by uniform count-normalized averaging over overlapping predictions:

(11) $$P^{\left(1\right)}\left(u,v\right)=\frac{\sum_{\left(x,y\right)\in \Omega \left(u,v\right)}p_{x,y}^{\left(1\right)}\left(u-y,v-x\right)}{\left|\Omega \left(u,v\right)\right|}$$

where $\Omega \left(u,v\right)$ denotes the set of sliding windows covering pixel $\left(u,v\right)$. This overlap aggregation reduces patch-border discontinuities while preserving the native mammogram scale. The resulting $P^{\left(1\right)}\in {\left[0,1\right]}^{H\times W}$ is used as the initial full-mammogram segmentation output and later serves as the probability prior for the Stage 2 residual refinement model.

### 3.2. Stage 2: Probability-Guided Residual Refinement

**Figure 2:**
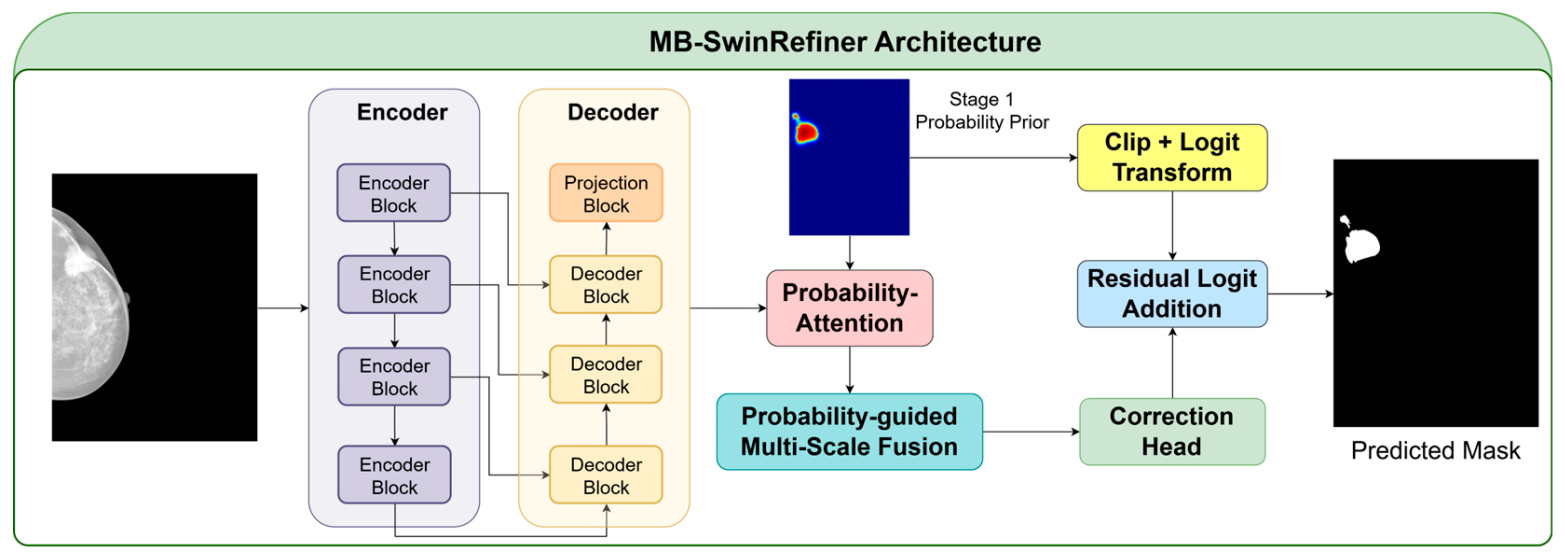
Stage 2 MB-SwinRefiner architecture. The aligned mammogram and Stage 1 probability map are used to predict an additive logit-space correction, producing the refined mask. Native setting: repeated for every aligned sliding-window coordinate.

The second stage of the proposed framework is MB-SwinRefiner (shown in Figure 2), a probability-guided residual refinement network designed to improve the initial Stage 1 prediction rather than re-segmenting the mammogram from scratch. Because MB-SwinRefiner follows the same general encoder–decoder reconstruction design described in Section 3.1, this section focuses only on the components that differ from MB-SwinUNet: the use of the Stage 1 probability map as an explicit prior, probability-guided decoder modulation, and logit-space residual correction.

Let $P^{\left(1\right)}$ denote the probability map generated by Stage 1. In the resized setting, Stage 1 directly produces a 512 × 512 probability map from the resized full mammogram. Stage 2 therefore receives one aligned image-level pair consisting of the complete resized mammogram X and its corresponding Stage 1 probability map $P^{\left(1\right)}$.

In the native sliding-window setting, Stage 1 first reconstructs a full-mammogram probability map at the original image resolution by aggregating overlapping patch predictions. Stage 2 then processes multiple aligned patch pairs rather than a single selected probability patch. For every sliding-window coordinate $c_{i}$, a mammogram patch $X_{i}$ and the spatially corresponding Stage 1 probability patch $P_{i}^{\left(1\right)}$ are extracted from the same coordinates.

During training, the corresponding ground-truth mask patch $Y_{i}$ is also extracted from the same coordinates. Each aligned pair $\left(X_{i},P_{i}^{\left(1\right)}\right)$ is independently passed through MB-SwinRefiner to produce a refined probability patch. During full-mammogram inference, all refined patches are returned to their original spatial locations, and predictions in overlapping regions are combined using count-normalized averaging. Thus, the native Stage 2 procedure is applied repeatedly across all sliding-window locations, whereas the resized Stage 2 procedure processes one complete resized image–probability pair.

MB-SwinRefiner uses the same Swin-Tiny encoder family and skip-connected convolutional decoder described for MB-SwinUNet. The encoder processes the mammogram image input, while the Stage 1 probability map is introduced as a guidance signal in the decoder. For every outer fold, the Stage 2 encoder was initialized using the corresponding selected outer-fold Stage 1 encoder weights; decoder and prediction-head parameters from Stage 1 are not transferred. This initialization provides mammography-specific feature representations while allowing the refiner to learn a separate correction function.

The main difference from Stage 1 is the probability-attention mechanism. At selected decoder levels, the aligned Stage 1 probability map is resized to the spatial size of the decoder feature and passed through a small convolutional gating module. For decoder level *l*, the probability gate is defined as

(12) $$G_{l}=\sigma \left(A_{l}\left(Resize\left(p_{p}^{\left(1\right)}\right)\right)\right),$$

where $A_{l}\left(\cdot \right)$ denotes the convolutional gating module and $\sigma \left(\cdot \right)$ is the sigmoid function. The decoder feature is then modulated using residual multiplicative gating:

(13) $${\hat{D}}_{l}^{\left(2\right)}=D_{l}^{\left(2\right)}⊙\left(1+\eta G_{l}\right),$$

where $D_{l}^{\left(2\right)}$ is the Stage 2 decoder feature at level l, η=0.1 is the attention strength, and ⊙ denotes element-wise multiplication. This formulation allows the Stage 1 probability prior to guide feature reconstruction without completely suppressing image-derived information. In the implemented configuration, probability attention is applied to the selected decoder levels before multi-scale decoder fusion.

After probability-guided modulation, MB-SwinRefiner uses the same multi-scale decoder fusion principle as Stage 1. The selected modulated decoder features are projected to a common channel dimension, aligned to the full 512×512 resolution, concatenated, and refined to produce the fused Stage 2 representation:

(14) $$F^{\left(2\right)}=R_{MSDF}\left(Concat\left({\tilde{D}}_{3}^{\left(2\right)},{\tilde{D}}_{2}^{\left(2\right)},{\tilde{D}}_{f}^{\left(2\right)}\right)\right),$$

The purpose of this fusion is different from Stage 1: instead of producing an initial segmentation representation, Stage 2 combines mammographic appearance features with probability-guided decoder features to estimate local corrections to the Stage 1 output.

The Stage 2 prediction head outputs a residual correction in logit space. Before Stage 2 training, the trained Stage 1 model is applied in inference mode to generate probability maps, which are stored and used as fixed inputs when constructing the Stage 2 dataset. Therefore, Stage 1 is not part of the Stage 2 computational graph, and no gradient is propagated through the stored probability maps. To prevent undefined or infinite values when a Stage 1 probability is numerically equal to 0 or 1, the probability patch is clipped before applying the logit function:

(15) $$z_{p}^{\left(1\right)}=logit\left(clip\left(p_{p}^{\left(1\right)},\epsilon ,1-\epsilon \right)\right),$$

where $\epsilon =10^{-4}$. The correction head predicts

(16) $$\Delta z_{p}=H_{corr}\left(F^{\left(2\right)}\right)$$

The refined Stage 2 logit is then computed by residual addition:

(17) $$z_{p}^{\left(2\right)}=z_{p}^{\left(1\right)}+\delta \Delta z_{p},$$

where δ controls the correction strength and is set to 1.0 in the implemented configuration. The refined probability output is obtained as

(18) $$p_{p}^{\left(2\right)}=\sigma \left(z_{p}^{\left(2\right)}\right).$$

This residual formulation makes the Stage 2 output explicitly depend on the Stage 1 belief. Rather than learning a new mask independently, the refiner learns how to modify the Stage 1 logit map where local evidence suggests that correction is needed. To stabilize this behavior, the final convolution of the correction head is initialized to zero, so that $\Delta z_{p}\approx 0$ at the start of training and the initial Stage 2 prediction is approximately equal to the Stage 1 prediction.

As in Stage 1, an auxiliary boundary head is attached to the fused representation during training. The boundary target is generated using the same morphological-gradient operation described in Section 3.1. The Stage 2 training objective is

(19) $$L_{stage2}={\omega_{1}L}_{focal}^{seg}+\omega_{2}L_{tversky}^{seg}+{\omega_{3}L}_{focal}^{bnd}$$

In the resized setting, the refined probability map is produced directly at 512×512 resolution and thresholded to obtain the final binary segmentation. In the native sliding-window setting, MB-SwinRefiner is applied to overlapping aligned mammogram and Stage 1 probability patches. The refined probability patches are written back to their original spatial locations and averaged over overlapping regions using the same count-normalized aggregation strategy described for Stage 1:

(20) $$P^{\left(2\right)}\left(u,v\right)=\frac{\sum_{\left(x,y\right)\in \Omega \left(u,v\right)}p_{x,y}^{\left(2\right)}\left(u-y,v-x\right)}{\left|\Omega \left(u,v\right)\right|}$$

The resulting $P^{\left(2\right)}$ is the final refined probability map of the two-stage framework. This design allows MB-SwinRefiner to operate consistently in both resized and native sliding-window settings, while preserving the central idea of probability-guided residual correction.

### 3.3. Dataset and Experimental Setup

Experiments were conducted on the INbreast full-field digital mammography dataset, which provides DICOM images and expert annotations [49]. The task was formulated as binary mass segmentation. The experimental index contained 410 mammograms from 108 cases: 107 images had target mass annotations and 303 were treated as target-negative. XML annotation files were available for 343 images, whereas 67 images had no annotation file. All processed mammograms were stored as 16-bit images.

Binary segmentation masks were generated from the XML annotations by labeling annotated masses as foreground and all remaining pixels as background. Target-negative mammograms were retained during training to improve background suppression. For comparability with previous INbreast mass segmentation studies, quantitative segmentation metrics were calculated only on the 107 mass-positive mammograms. Boundary targets for auxiliary supervision were derived from the binary masks using the morphological-gradient operation described in Section 3.1.

All experiments used stratified five-fold cross-validation at the case level to prevent data leakage and reduce split-dependent bias [50]. Cases were stratified according to whether they contained at least one mass-positive mammogram. In each outer fold, one fold was reserved for testing, while 10% of the remaining cases were randomly assigned to validation and the rest to training. All mammograms from the same case remained in the same subset. The validation set was used for checkpoint and segmentation-threshold selection, and the test fold remained excluded from all training and model-selection procedures. Identical case-level splits were used for Stages 1 and 2.

To avoid training Stage 2 with in-sample Stage 1 predictions, five-fold inner cross-fitting was performed within each outer training subset. For each inner fold, Stage 1 was trained on the remaining inner folds and applied only to the held-out cases. Repeating this procedure produced out-of-fold probability maps for every outer-training case, which were used to construct the Stage 2 training set. The Stage 1 model selected using the outer validation set was then applied separately to the outer validation and test cases to generate fixed probability maps. Stage 2 was trained on the out-of-fold training maps, selected on the validation set, and evaluated only on the held-out test fold.

Two experimental protocols were considered. In the resized setting, each mammogram and mask were resized to 512 × 512. Stage 1 predicted the segmentation directly from the resized mammogram, and Stage 2 received the aligned mammogram and Stage 1 probability map as inputs. This protocol enabled contextual comparison with previous INbreast studies using resized full-mammogram images.

In the native sliding-window setting, Stage 1 was trained on 512 × 512 patches extracted at the original mammogram resolution. Patches were sampled only from training cases and included dense-background, breast-background, mass-boundary, near-mass negative, and mass-core regions to address foreground sparsity while maintaining sufficient negative examples. During inference, overlapping windows with a stride of 256 pixels were applied across the full mammogram, and patch probabilities were combined by count-normalized averaging. For Stage 2, the mammogram patch, Stage 1 probability patch, and ground-truth mask patch were extracted from identical coordinates. Refined predictions were aggregated using the same sliding-window procedure, preserving both native image scale and spatial alignment between the image and probability prior.

**Preprocessing and augmentation.** Mammograms were horizontally reoriented when necessary so that the chest wall was on the left and the breast extended toward the right. The 16-bit intensities were divided by 65,535 and normalized using a mean of 0.5 and a standard deviation of 0.25. During training, random affine transformation was applied with probability 0.3, including translation up to 4%, scaling within 0.92–1.08, and rotation within ±10°. Brightness and contrast adjustments within ±0.1 were applied with probability 0.3, and gamma adjustment within 0.85–1.15 was applied with probability 0.15. Horizontal flipping was disabled, and no augmentation was applied during validation or testing. Table 2 summarizes the main training and inference settings used in the experiments.

### 3.4. Evaluation Metrics

Segmentation performance was evaluated using the Dice similarity coefficient (*DSC*), intersection over union (*IoU*), sensitivity, and specificity. These metrics were computed from pixel-level true positives (*TP*), true negatives (*TN*), false positives (*FP*), and false negatives (*FN*). *DSC* and *IoU* were used to measure the overlap between the predicted binary mask and the ground-truth mass mask, while sensitivity and specificity were used to assess foreground detection and background suppression, respectively. Metrics were calculated separately for each held-out mass-positive mammogram. Within each outer test fold, the image-level scores were averaged across the mass-positive mammograms, and the reported cross-validation results were calculated as the mean and standard deviation of the fold-level averages across the five outer folds. The metrics were defined as follows:

(21) $$DSC=\frac{2TP}{2TP+FP+FN}$$

(22) $$IoU=\frac{TP}{TP+FP+FN}$$

(23) $$Sensitivity=\frac{TP}{TP+FN}$$

(24) $$Specificity=\frac{TN}{TN+FP}$$

## 4. Experimental Results

### 4.1. Resized 512 × 512 Benchmark Comparison

The first experimental setting evaluates the proposed framework under a resized image-level protocol. This setting was included because several previously reported INbreast mammographic mass segmentation studies use resized mammographic inputs, making it the most suitable setting for contextual comparison with existing methods. In this protocol, each full mammogram and its corresponding ground-truth mask were resized to 512×512. Stage 1 MB-SwinUNet generated an initial probability map at the resized image resolution, and Stage 2 MB-SwinRefiner refined this prediction through probability-guided residual correction at the same spatial resolution.

Table 3 presents the ablation study and stage-wise performance of the proposed framework in the resized setting. The complete Stage 1 model, which uses both multi-scale decoder fusion and training-only boundary supervision, achieved Dice of 85.70 ± 2.90%, IoU of 84.07 ± 2.60%, sensitivity of 87.40 ± 3.50%, and specificity of 99.97 ± 0.02%. Removing the auxiliary boundary branch while keeping MSDF enabled reduced Dice to 83.36 ± 3.30% and IoU to 82.54 ± 2.90%, indicating that contour-derived auxiliary supervision improves region-overlap performance in the resized setting. Removing MSDF caused a larger reduction, decreasing Dice to 78.22 ± 3.40% and IoU to 76.42 ± 3.50%, which supports the importance of combining reconstructed decoder representations before final mask prediction.

The final configuration corresponds to the complete two-stage framework with probability-guided residual refinement. Compared with the complete Stage 1 model, MB-SwinRefiner improved Dice from 85.70 ± 2.90% to 86.19 ± 3.00% and IoU from 84.07 ± 2.60% to 84.33 ± 2.80%. Sensitivity increased from 87.40 ± 3.50% to 89.34 ± 3.30%, while specificity remained high, increasing from 99.97 ± 0.02% to 99.98 ± 0.01%. These results suggest that probability-guided residual refinement provides a modest improvement over the Stage 1 probability map in the resized benchmark setting, particularly in sensitivity.

Table 4 compares the proposed resized models with previously reported INbreast mammographic mass segmentation results obtained under resized image-level settings. The compared methods include single-stage resized segmentation models and presegmenter cascaded frameworks. Among the prior methods listed, Cas-DeepLabV3+ with ResNet50 reported the highest Dice score, with Dice of 80.00%, sensitivity of 78.00%, and specificity of 99.88%. In comparison, the proposed MB-SwinUNet achieved Dice of 85.70%, sensitivity of 87.40%, and specificity of 99.97%, while the full MB-SwinRefiner framework achieved Dice of 86.19%, sensitivity of 89.34%, and specificity of 99.98%.

Overall, the resized benchmark results provide the most direct comparison with previously reported INbreast studies using resized mammographic inputs. However, because published studies may still differ in data partitioning, preprocessing, augmentation, threshold selection, and metric aggregation, Table 4 should be interpreted as a contextual benchmark comparison rather than a fully controlled head-to-head evaluation.

### 4.2. Native Sliding-Window Extension

Table 5 presents the native sliding-window ablation and stage-wise refinement results. In this setting, overlapping patch predictions were aggregated to reconstruct full-mammogram probability maps at the original image scale. Compared with the resized benchmark, this protocol introduces additional challenges related to foreground sparsity, patch consistency, and false responses across a large anatomical background.

Among the native Stage 1 configurations, the complete MB-SwinUNet achieved the best mean overlap performance, with Dice of 76.64 ± 5.40% and IoU of 74.38 ± 6.70%. Removing auxiliary boundary supervision while retaining multi-scale decoder fusion reduced Dice to 76.17 ± 6.10% and IoU to 73.89 ± 6.90%, indicating a small but consistent benefit from contour-derived supervision. In contrast, removing MSDF caused a substantially larger decrease, reducing Dice to 61.54 ± 7.20% and IoU to 58.67 ± 5.80%. These findings identify multi-scale decoder fusion as the principal contributor to native Stage 1 performance.

The complete native two-stage framework further improved Dice to 78.06 ± 5.90% and IoU to 76.11 ± 6.40%, corresponding to gains of 1.42 and 1.73 percentage points over Stage 1, respectively. These results indicate that probability-guided residual refinement provides a modest improvement after full-image sliding-window reconstruction and supports its role in correcting local errors in the initial probability map.

### 4.3. Computational Complexity

Table 6 summarizes the parameter count and computational complexity of Stage 1 MB-SwinUNet and Stage 2 MB-SwinRefiner. FLOPs were measured using the PyTorch v2.13 profiler for a single 512 × 512 input with batch size 1. Stage 1 contains 32.150 million parameters and requires 246.154 GFLOPs per forward pass, whereas Stage 2 contains 32.213 million parameters and requires 252.855 GFLOPs.

### 4.4. Case-Level Paired Analysis in the Resized Setting

Patient-level paired analysis showed that Stage 2 improved all evaluated segmentation metrics compared with Stage 1 (see Table 7). Mean Dice increased by 4.62 percentage points (95% CI, 0.54–9.79; two-sided Wilcoxon signed-rank *p* < 0.001), with higher Dice observed in 47 of 50 cases and lower Dice in 3 cases. Mean IoU increased by 4.39 percentage points (95% CI, 0.31–9.55; *p* < 0.001), sensitivity by 2.76 percentage points (95% CI, 2.01–3.33; *p* < 0.001), and specificity by 0.014 percentage points (95% CI, 0.010–0.017; *p* < 0.001). These results indicate a statistically significant patient-level refinement benefit, although the magnitude of improvement varied across metrics.

## 5. Discussion

This study proposed MB-SwinRefiner, a two-stage framework for mammographic mass segmentation that combines Swin-based contextual encoding, multi-scale decoder fusion, auxiliary boundary supervision, and probability-guided residual refinement. The framework was evaluated on INbreast using case-level five-fold cross-validation under two complementary protocols: resized 512 × 512 image-level segmentation and native sliding-window full-mammogram reconstruction. Overall, Stage 2 improved the initial Stage 1 predictions, while the resized results provided a competitive contextual comparison with previously reported INbreast methods.

In the resized setting, MB-SwinUNet achieved Dice of 85.70 ± 2.90%, IoU of 84.07 ± 2.60%, sensitivity of 87.40 ± 3.50%, and specificity of 99.97 ± 0.02%. After probability-guided refinement, MB-SwinRefiner achieved Dice of 86.19 ± 3.00%, IoU of 84.33 ± 2.80%, sensitivity of 89.34 ± 3.30%, and specificity of 99.98 ± 0.01%. The modest Dice and IoU gains indicate that Stage 1 already provided strong overlap performance, whereas the larger sensitivity improvement suggests that Stage 2 recovered lesion pixels that were missed or under-segmented initially. Such improved lesion coverage may be useful for downstream analysis, although diagnostic performance and clinical utility were not evaluated.

The results reflect the complementary roles of the proposed components. The Swin encoder provides hierarchical context for distinguishing masses from dense fibroglandular tissue, while multi-scale decoder fusion combines coarse lesion extent with intermediate and fine spatial information. Auxiliary boundary supervision encourages contour-sensitive representation learning without adding an inference-time branch. Stage 2 differs from an independent replacement model because the fixed Stage 1 probability map guides decoder reconstruction and the output head predicts an additive correction to the Stage 1 logits.

**Figure 3:**
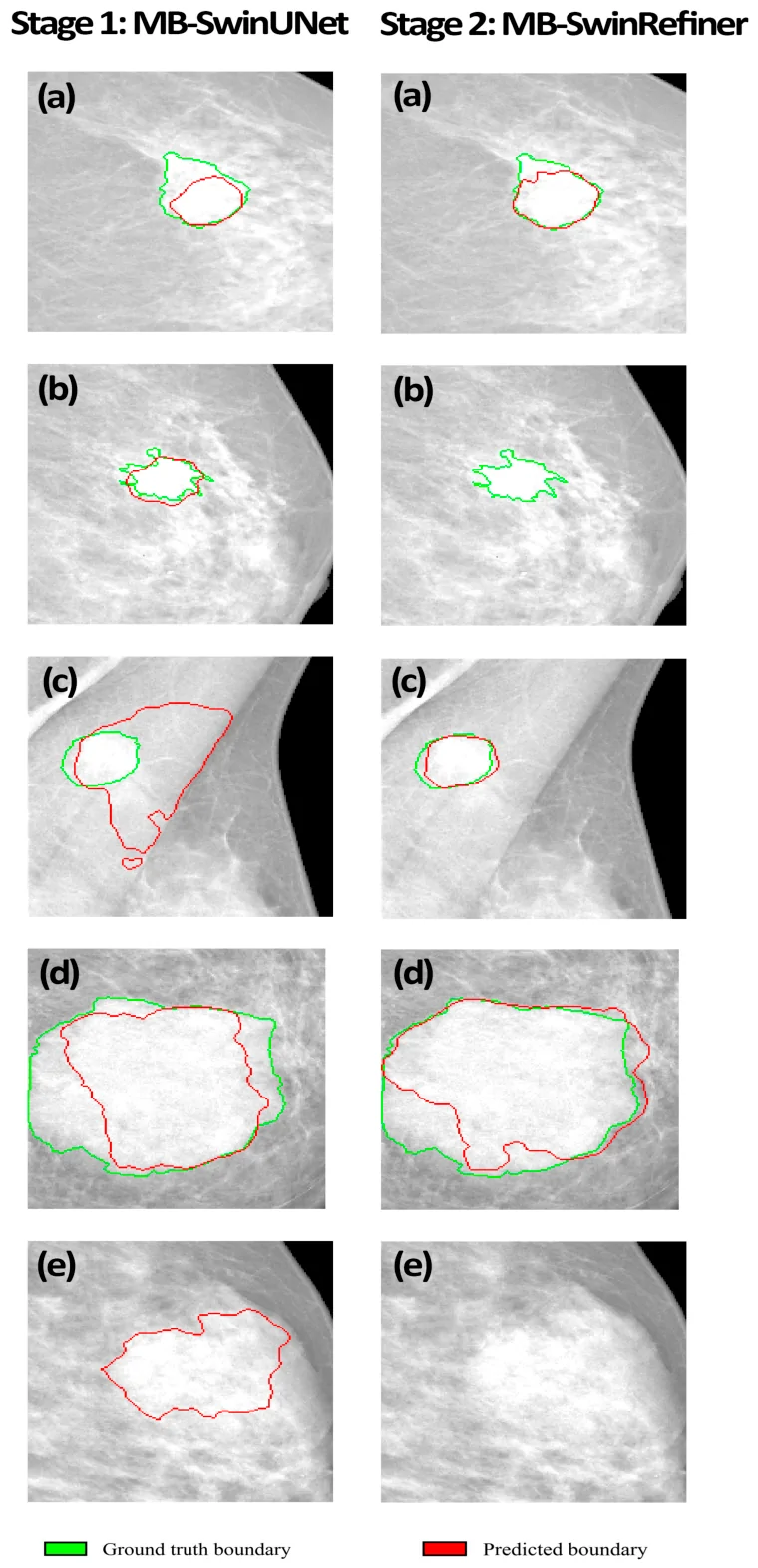
Qualitative comparison of segmentation performance between Stage 1 (MB-SwinUNet) and Stage 2 (MB-SwinRefiner) on representative cases from the INbreast dataset (**a**–**e**). Green contours denote the ground truth boundaries, while red contours represent the predicted boundaries of segmented masses. MB-SwinRefiner demonstrates strong segmentation quality in cases (**a**,**c**–**e**). However, it exhibits a failure to segment the mass in case (**b**).

**Figure 4:**
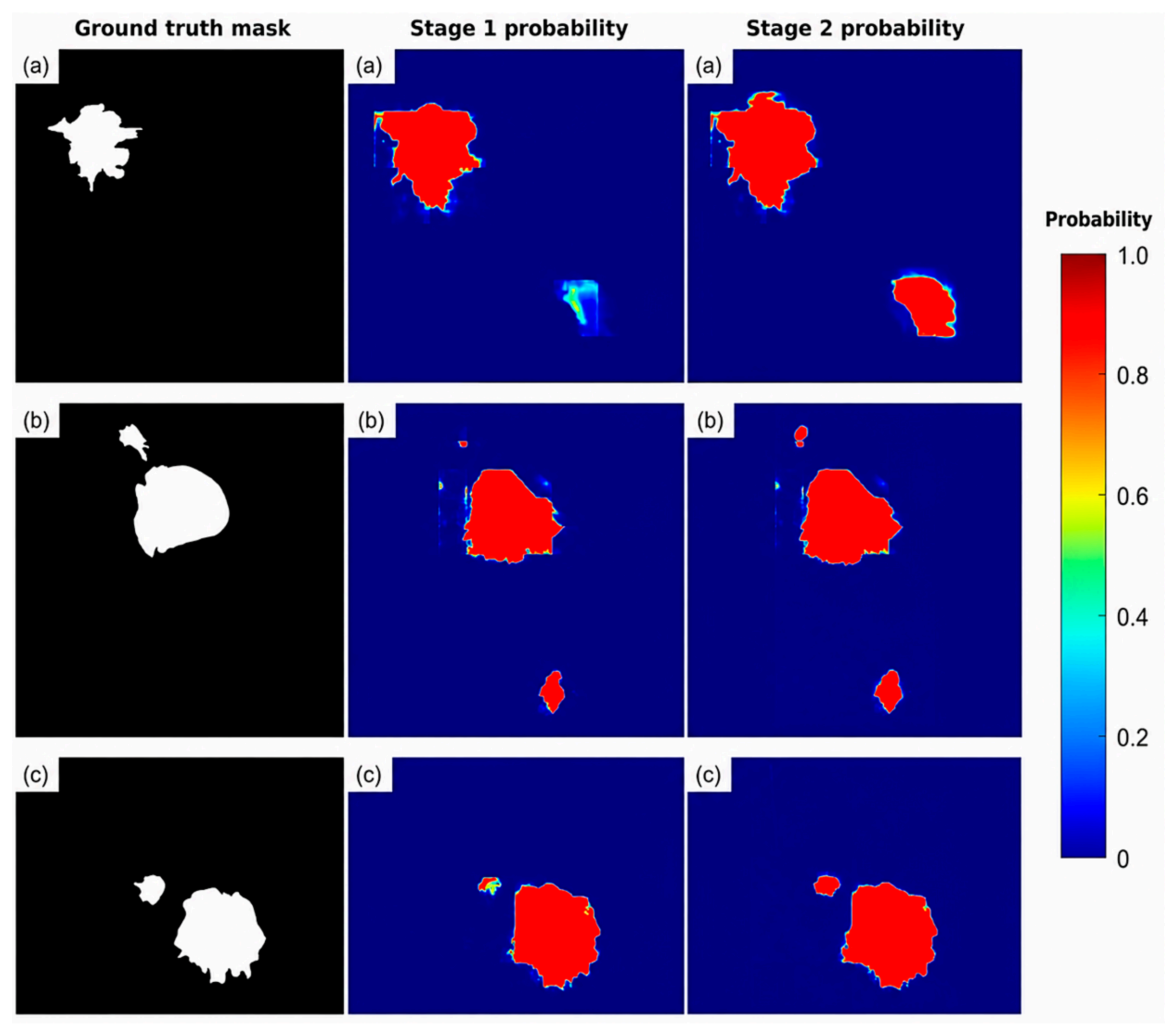
Qualitative visualization of native sliding-window probability maps for three representative INbreast test cases (**a**–**c**). Each row presents, from left to right, the ground-truth mask, the raw probability map from Stage 1 (MB-SwinUNet), and the refined probability map from Stage 2 (MB-SwinRefiner), all shown prior to thresholding. Stage 2 successfully enhances the probability values across the target regions in cases (**b**,**c**); conversely, in case (**a**), the Stage 2 results in a comparatively weaker probability response.

The qualitative findings in Figure 3 and Figure 4 provide additional insight into this refinement behavior. In several resized cases, Stage 2 improved incomplete lesion coverage and reduced over-segmentation into surrounding parenchyma. It also suppressed a Stage 1 false-positive response in one target-negative example, although a dedicated lesion-level false-positive analysis was not performed. In the native examples, Stage 1 probability maps sometimes showed incomplete coverage, reduced confidence near irregular margins, or patch-like patterns resulting from sliding-window reconstruction. Stage 2 generally increased probability values across the target region and improved lesion coverage, consistent with the native quantitative results. However, refinement occasionally preserved or amplified small non-lesion responses and, in one example, over-corrected a weak initial prediction and suppressed the lesion. These observations indicate that the refiner remains dependent on the quality of the Stage 1 probability map, particularly for small, weakly contrasted, or low-confidence lesions. False-positive control and patch-boundary consistency therefore remain important challenges.

The ablation results support the principal architectural choices. Removing auxiliary boundary supervision reduced resized Dice from 85.70% to 83.36%, indicating that contour-derived supervision provided useful training guidance. Removing multi-scale decoder fusion produced a larger reduction, decreasing Dice to 78.22% and IoU to 76.42%, suggesting that fusion of reconstructed decoder features was a major contributor to Stage 1 performance. In the native setting, Stage 2 improved mean Dice from 76.64% to 78.06% and mean IoU from 74.38% to 76.11%, showing that residual refinement remained beneficial when full-image predictions were reconstructed from overlapping patches. The lower native performance relative to resized evaluation is expected because native inference must address foreground sparsity, patch consistency, extensive anatomical background, and false responses across the full mammogram.

In the contextual comparison with previously reported resized INbreast methods, Cas-DeepLabV3+ with ResNet50 reported the highest previous Dice of 80.00%, whereas MB-SwinUNet and MB-SwinRefiner achieved 85.70% and 86.19%, respectively. The proposed models also showed higher sensitivity than the listed methods, suggesting improved lesion-pixel recovery. Nevertheless, these comparisons should be interpreted cautiously because published studies may differ in preprocessing, data partitioning, augmentation, threshold selection, and metric aggregation. The comparison therefore provides contextual evidence rather than a controlled head-to-head evaluation.

This study has several limitations. First, evaluation was restricted to the relatively small INbreast dataset, and external validation on larger and more diverse mammography datasets is required. Second, although case-level five-fold cross-validation reduces split-dependent bias, protocol differences continue to limit direct comparison with previous studies. Third, the native sliding-window experiment has limited comparability with existing literature and should be interpreted primarily as an internal full-resolution analysis. Fourth, the evaluation focused on pixel-level region metrics and did not include dedicated boundary, lesion-level detection, false-positive, or downstream clinical-utility analyses. Finally, the Stage 2 comparison evaluated the complete refinement framework without independently separating the effects of probability guidance, residual prediction, encoder initialization, and additional model capacity. The findings should therefore be interpreted as internal segmentation results on INbreast rather than evidence of cross-dataset generalizability or clinical effectiveness. Future work should include factorized refinement ablations, boundary and lesion-level metrics, lesion-subtype analysis, false-positive evaluation, and external validation under diverse acquisition conditions.

## 6. Conclusions

This paper proposes MB-SwinRefiner, a two-stage mammographic mass segmentation framework that leverages an initial probability map to guide residual refinement for the challenging task of breast mass segmentation. The probability map generated by the first-stage MB-SwinUNet model is used as prior information in the second-stage refinement model, allowing MB-SwinRefiner to focus on correcting local segmentation errors rather than re-segmenting the mammogram independently. The proposed framework combines Swin-based contextual encoding, multi-scale decoder fusion, training-only contour-derived supervision, and probability-guided residual correction to improve region-based mass segmentation and probability-map refinement.

The proposed framework was validated on the INbreast dataset under both resized benchmark evaluation and native sliding-window full-mammogram analysis. The internal cross-validation results show a modest improvement in the evaluated segmentation metrics after probability-guided refinement compared with the first-stage model. The main contribution of this study is the residual refinement mechanism, which uses the Stage 1 probability map as guidance for correcting local segmentation errors and refining full-mammogram predictions.

## Figures and Tables

**Table 1:** Positioning of the proposed method relative to representative mammographic and medical segmentation approaches.

Category	Representative Methods	Main Idea	Representative Gap Relevant to This Work
CNN-based	Attention Dense-U-Net, AUNet, DCANet, ARF-Net	Local-to-regional feature learning, attention, affinity, adaptive receptive field	Limited explicit long-range contextual modeling under dense-tissue ambiguity
Reconstruction/skip-enhanced	FS-UNet, Connected-UNets, Connected-SegNets, EU-Net	Improved feature transfer, cascaded reconstruction, enhanced U-shaped decoding	Final prediction may still depend on sequential decoder reconstruction
Transformer/hybrid mammographic models	YOLO-LOGO, TrEnD, Swin-SFTNet, HTU-Net, Mammo-SAM	Stronger contextual encoding, hybrid fusion, task-specific foundation-model adaptation	Pixel-level boundary recovery still depends on decoder and refinement strategy
General decoder-centric	UNet++, UNet 3+, EMCAD	Nested/full-scale skip aggregation and multi-scale decoding	Fusion is not specifically focused on reconstructed decoder-stage hypotheses before final prediction
Boundary-aware	Boundary Loss, Hausdorff loss, Gated-SCNN, BEA-Net	Boundary-sensitive loss or persistent edge/shape streams	May introduce extra inference pathways or does not target mammographic native-scale refinement

**Table 2:** Experimental training and inference settings.

Setting	Value
Input patch size	512×512
Number of epochs	100
Encoder backbone	Swin-Tiny (4×4 patch embedding; 7×7 attention window)
Boundary target	Morphological gradient, 10×10 kernel
Optimizer	AdamW
Initial learning rate	3 × 10^−4^
Weight decay	1 × 10^−4^
Learning-rate scheduler	Cosine annealing
Batch size	8
Threshold selection	Validation sweep selected by Dice
Segmentation loss	Focal + Tversky
Boundary loss	Focal
Inference strategy	sliding-window
Inference stride	256
Focal loss	α=0.8, γ=2.0, $\omega_{1}=1.0$
Tversky loss	α=0.3, β=0.7, smooth=1.0, $\omega_{2}=1.0$
Boundary focal loss	α=0.9, γ=2.0, $\omega_{3}=2.0$
Random seed	42

**Table 3:** Ablation study and stage-wise performance of the proposed framework in the resized 512 × 512. Bold values indicate the best result for each performance metric.

Model/Evaluation Setting	MSDF	Boundary Supervision	Residual	Dice (%)	IoU (%)	Sensitivity (%)	Specificity (%)
Stage 1—resized	On	On	Off	85.70 ± 2.9	84.07 ± 2.6	87.40 ± 3.5	99.97 ± 0.02
Stage 1—resized	On	Off	Off	83.36 ± 3.3	82.54 ± 2.9	85.14 ± 3.6	99.96 ± 0.02
Stage 1—resized	Off	On	Off	78.22 ± 3.4	76.42 ± 3.5	81.37 ± 3.1	99.95 ± 0.03
**Stage 2—resized**	**On**	**On**	**On**	**86.19 ± 3.0**	**84.33 ± 2.8**	**89.34 ± 3.3**	**99.98 ± 0.01**

**Table 4:** Comparison with resized 512 × 512 INbreast mammographic mass segmentation results. Bold values indicate the best result for each performance metric.

Method	Dice (%)	Sensitivity (%)	Specificity (%)	Framework Type
Attention U-Net [51]	65.00	65.00	99.94	Single-stage resized segmentation
Swin Transformer [51]	63.00	60.00	99.80	Single-stage resized segmentation
DeepLabV3+ with ResNet50 [51]	77.00	78.00	99.86	Single-stage resized segmentation
Cas-Attention U-Net [51]	71.00	71.00	99.80	Presegmenter cascaded framework
Cas-Swin Transformer [51]	68.00	71.00	99.68	Presegmenter cascaded framework
Cas-DeepLabV3+ with ResNet50 [51]	80.00	78.00	99.88	Presegmenter cascaded framework
MB-SwinUNet	85.70	87.40	99.97	Resized Stage 1 segmentation
**MB-SwinRefiner**	**86.19**	**89.34**	**99.98**	Resized Stage 2 segmentation

**Table 5:** Native sliding-window ablation and stage-wise refinement performance of the proposed framework. Bold values indicate the best result for each performance metric.

Model/Evaluation Setting	MSDF	Boundary Supervision	Residual	Dice (%)	IoU (%)
Native sliding-window—Stage 1	On	On	Off	76.64 ± 5.4	74.38 ± 6.7
Native sliding-window—Stage 1	On	Off	Off	76.17 ± 6.1	73.89 ± 6.9
Native sliding-window—Stage 1	Off	On	Off	61.54 ± 7.2	58.67 ± 5.8
**Native sliding-window—Stage 2**	**On**	**On**	**On**	**78.06 ± 5.9**	**76.11 ± 6.4**

**Table 6:** Computational characteristics of Stage 1 and Stage 2 for a single 512 × 512 input with batch size 1 using FP32 precision. Throughput and GPU memory were measured on an NVIDIA GeForce GTX 1660 SUPER GPU (ASUSTeK Computer Inc., Taipei, Taiwan).

Model	Parameters (M)	GFLOPs	img/s	Model Memory (MiB)
MB-SwinUNet	32.150	246.154	12.10	127.86
MB-SwinRefiner	32.213	252.855	11.11	128.10

**Table 7:** Patient-level paired comparison of Stage 1 and Stage 2 performance in the resized setting. Values are reported as mean ± standard deviation across 50 mass-positive cases. Differences were calculated as Stage 2 minus Stage 1 and are expressed in percentage points. The 95% confidence intervals were estimated using a fold-stratified paired bootstrap with 10,000 resamples. *p*-values were obtained using two-sided Wilcoxon signed-rank tests.

Metric	Stage 1 (%)	Stage 2 (%)	Mean Difference (pp)	95% CI (pp)	Wilcoxon (p)
Dice	83.59 ± 16.84	88.21 ± 5.84	+4.62	0.54 to 9.79	<0.001
IoU	81.96 ± 16.79	86.35 ± 5.73	+4.39	0.31 to 9.55	<0.001
Sensitivity	86.82 ± 4.95	89.58 ± 4.73	+2.76	2.01 to 3.33	<0.001
Specificity	99.968 ± 0.020	99.982 ± 0.013	+0.014	0.010 to 0.017	<0.001

## Data Availability

The data that support the findings of this study are from the INbreast dataset (Moreira et al., [49] at https://doi.org/10.1016/j.acra.2011.09.014), which is publicly accessible at: https://www.kaggle.com/datasets/ramanathansp20/inbreast-dataset (accessed on 2 August 2026).
